# Investigation of Conserved Amino Acids in the EGL-15 5A Domain Required for Sex Myoblast Migration in *C. elegans*

**DOI:** 10.17912/micropub.biology.001374

**Published:** 2024-10-15

**Authors:** Birsen Gürkaynak, Dallas R. Fonseca, Isabella Suarez, Michael J. Stern, Te-Wen Lo

**Affiliations:** 1 Biology, Ithaca College, Ithaca, New York, United States; 2 Research Department, New England Biolabs (United States), Ipswich, Massachusetts, United States; 3 Biology, Northeastern Illinois University, Chicago, Illinois, United States

## Abstract

Fibroblast Growth Factor Receptors (FGFRs) play a role in diverse developmental pathways that mediate cell proliferation, cell migration, and cell survival. We use *C. elegans* as a model to better understand FGFR signaling specificity. *C. elegans* has a single FGFR, EGL-15 . EGL-15 contains an alternatively spliced exon 5 that results in two isoforms, EGL-15(5A) and EGL-15(5B), that differ in their extracellular domains. The EGL-15(5A) isoform is required for the chemoattraction of a pair of migrating sex myoblasts, whose correct positioning is required for egg laying. Deletion of the 5A domain results in an egg-laying defective (Egl) organism. We identified six highly conserved amino acids within the 5A domain and found that two amino acids, S145 and L148 are specifically required for sex myoblast migration in *C. elegans* .

**Figure 1. EGL-15 5A Domain Analysis:**
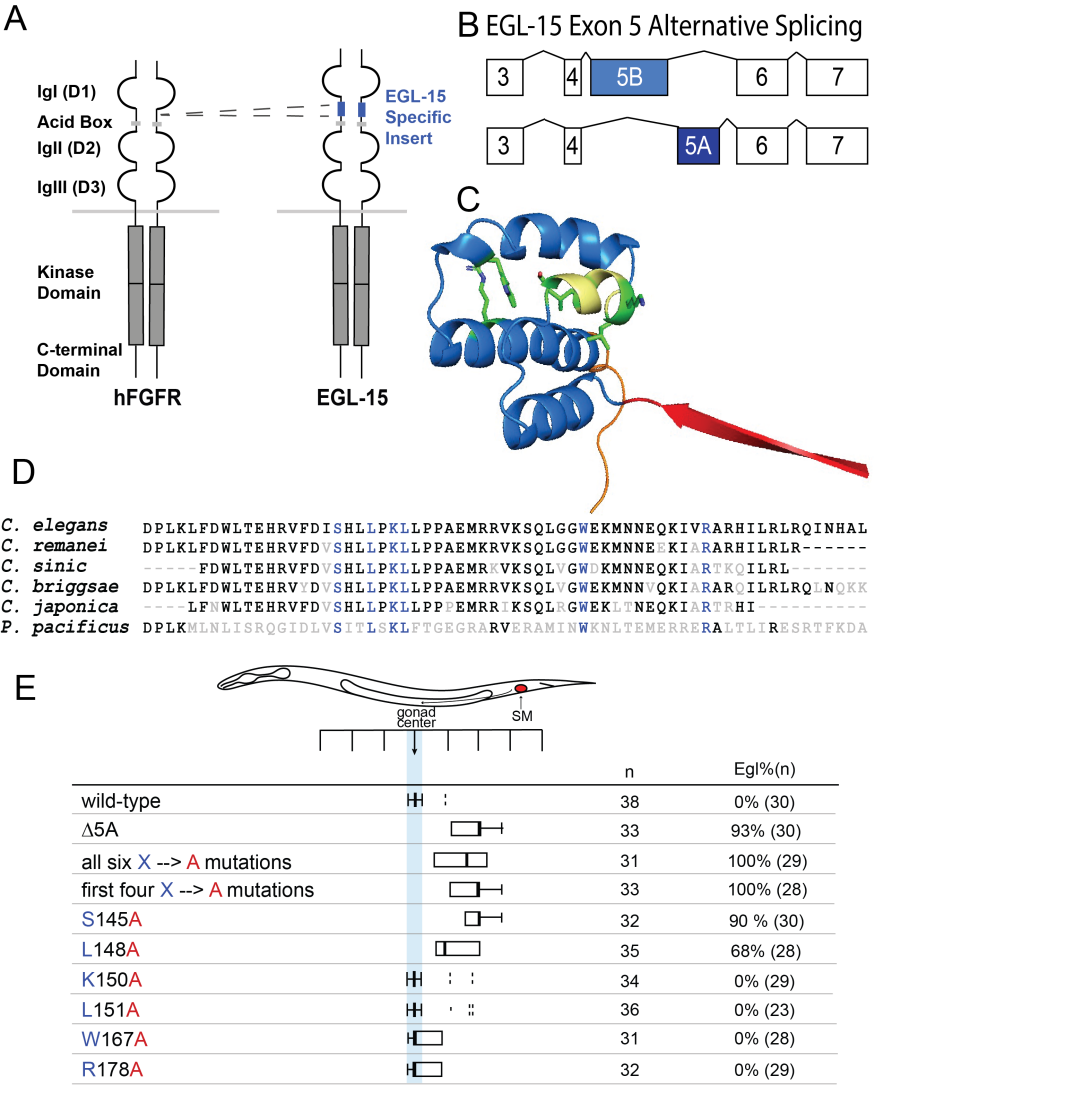
A) Structural comparison of the human FGFR and the worm FGFR(EGL15). EGL-15 contains an EGL-15-specific insert. (B) Alternative splicing of *egl-15* exon 5. *egl-15* exon five is alternatively spliced to give rise to an EGL-15 5A isoform and an EGL-15 5B isoform. (C) EGL-15 5A domain structure modeled using AlphaFold and visualized using PyMOL software, version 2.5.2. The 5A domain is colored blue (D128- L192). The 10 amino acids N-terminal to the 5A domain are colored red, the ten C-terminal amino acids orange, and the rest of the EGL-15 protein is hidden. The region of the four clustered conserved amino acids that show effects on SM migration is colored yellow, with the six conserved amino acids colored green and the structures of their R-groups highlighted. The conserved amino acids, ordered in space are: R178 – W167 – S145/L148 – L151 – K150. (D) EGL-15 5A domain alignment. Six conserved amino acids are shown in blue. (E) Sex myoblast position and Egl phenotype analysis.

## Description

Fibroblast growth factor receptors (FGFRs), a subfamily of Receptor Tyrosine Kinases (RTKs), are responsible for mediating various biological events including cell proliferation, angiogenesis, differentiation, cell migration, and cell survival (Itoh and Ornitz, 2011; Ornitz and Itoh, 2015) . FGFRs are highly conserved. In humans, signaling specificity is achieved through a complex matrix of regulatory mechanisms and interactions between a total of four FGFRs and 18 FGFs (Itoh and Ornitz, 2011) . To circumvent this complexity, *C. elegans* can be used as a model organism for understanding FGFR signaling specificity. The sole *C. elegans* FGFR, EGL-15 , is known to mediate several distinct processes, including the chemoattraction of the two myoblast precursors of the egg-laying muscles (sex myoblasts) (Sherwood and Plastino, 2018) . In wild-type, the sex myoblasts (SMs) are born in the posterior of *C. elegans* and migrate anteriorly during the early larval stages to flank the precise center of the gonad, where they divide and differentiate to give rise to all of the egg-laying muscles (Sulston and Horvitz, 1977) . Failure of the sex myoblasts to migrate properly results in an Egl (egg-laying defective) phenotype (Trent et al., 1983) . The *C. elegans* FGFR, EGL-15 is structurally similar to the human FGFR, however, there is a unique domain present between the first IgG domain and the acid box domain, termed the EGL-15 specific insert. The EGL-15 specific insert is encoded for completely by exon 5 and is alternatively spliced to give rise to the 5A isoform (containing the 5A domain) and the 5B isoform (containing the 5B domain) (Goodman et al., 2003) . Deletion of both domains, or just the 5A domain, results in an Egl phenotype due to posteriorly displaced sex myoblasts. Deletion of the 5B domain alone does not result in a visible phenotype and to date, a specific role of the 5B domain has yet to be identified (Lo et al., 2008) .

Given the known role for the 5A domain in sex myoblast migration (Lo et al., 2008) , we employed an evolutionary approach to identify amino acids within the 5A domain that are required for proper sex myoblast migration. Alignment of the 65 amino acid 5A domain in *C. elegans* with five other nematode species, revealed six amino acids which are conserved among all of the six species, including *Pristionchus pacificus* , which is more evolutionarily distant than the other five. Four of these six conserved amino acids are clustered together at the N-terminal end of the 5A domain. Mutants strains were generated using CRISPR (through SUNYbiotech) containing either (1) deletion of the entire 5A domain (Δ5), (2) all six amino acids mutated to alanines (all six), (3) the first four conserved amino acids mutated to alanines (first four), or (4) each single conserved amino acid altered to alanine. The final positions of the sex myoblasts in each of these strains were examined, revealing a role for several exon 5A amino acids in sex myoblast migration ( Figure 1E ). Consistent with previous data generated using transgenes (Lo et al., 2008) , removal of the entire 5A domain results in posteriorly displaced sex myoblasts.

Mutation of all six conserved amino acids mimics the effect on SM positioning similar to the Δ5 mutant, however with a broader distribution of sex myoblasts that are more anteriorly positioned (Tukey analysis, p< 0.0001), suggesting a potential structural role for the entire 5A domain. Alteration of the first four conserved amino acids had a similar distribution as the Δ5 domain (Turkey analysis, p>0.05) and the single S145A mutant (Turkey analysis, p>0.05), suggesting that S145A plays a critical role in the positioning of sex myoblasts. Interestingly, the L148A mutation also resulted in slightly posteriorly-displaced sex myoblasts. When sex myoblast positions were compared between the L148A mutant and the mutant with all six conserved amino acids mutated, there was no significant difference (Tukey analysis, p>0.05), but there was a significant difference when compared to sex myoblasts in the S145A mutant (Tukey analysis, p<0.001) and the first four amino acid mutant (Tukey analysis, p<0.001). Three-dimensional modeling in Alphafold suggest the possibility that the S145 and L148 may define a critical point of interaction based on their positioning within the suggested model ( Figure 1C ). K150A and L151A single mutations did not appear to effect sex myoblast positioning. W167A and R178A single mutations resulted in properly centered sex myoblasts, but with larger distributions than wild-type. Taken together, this analysis lends further support for important informational content in the 5A domain for SM chemoattraction, and a critical role for S145 and L148.

## Methods

*Strain Maintenance*

N2 strain was obtained from the Liu Lab at Cornell University. *egl-15* (5A domain) mutant strains were obtained from SUNYbiotech. Strains were grown and maintained according to Wormbook (Stiernagle, 2006) .

*mCherry strain construction*

To more easily visualize sex myoblast positions, *egl-15* (5A) domain mutants were crossed to LW3949 ( jjIs3900 [srsIm15.129pJKL1066.3:hlh-8p::nls::mCherry::lacZ]+ myo-2 ::mCherry]) to generate strains that contained both the *egl-15* (5A) domain mutation and sex myoblasts expressing mCherry using standard genetic methods.

*Sex Myoblast Position Analysis*

Each strain was bleached to obtain a synchronized population of embyros. Embryos were plated on NGM plates with OP50 and allowed to develop at 20°C until they reached the mid-late L3 stage. Gonad development was used as a crude marker. Sex myoblast positions were recorded when the gonad had turned. Sex myoblast positions were determined with respect to the underlying hypodermal Pn.P cells (Thomas et al., 1990) and represent using box-and-whisker plots aligned to a Pn.p “ruler.”

*Egl Phenotype Quantification*

For each strain, 30 L4 hermaphrodites were picked to a single plate and allowed to develop for 24 hours at 20°C. After 24 hours, individual worms were scored as Egl or non-Egl. Worms that possessed more than a single row of embryos in the uterus were scored as Egl.

## Reagents

**Table:** 

Strain	Genotype	Available From
N2	*Caenorabditis elegans*	CGC
PHX6504	D5A *egl-15 ( syb6504 )X *	SUNYbiotech
PHX6343	all six *egl-15 ( syb6343 )X *	SUNYbiotech
PHX6294	first four *egl-15 ( syb6294 )X *	SUNYbiotech
PHX6346	S145A *egl-15 ( syb6346 )X *	SUNYbiotech
PHX6335	L148A *egl-15 ( syb6335 )X *	SUNYbiotech
PHX6438	K150A *egl-15 ( syb6438 )X *	SUNYbiotech
PHX6431	L151A *egl-15 ( syb6431 )X *	SUNYbiotech
PHX6350	W167A *egl-15 ( syb6350 )X *	SUNYbiotech
PHX6624	R178A *egl-15 ( syb6624 )X *	SUNYbiotech
LW3949	*jjIs3900 [srsIm15.129pJKL1066.3:hlh-8p::nls::mCherry::lacZ]+ myo-2 ::mCherry]IV *	Liu Lab
TWL33	*jjIs3900 [srsIm15.129pJKL1066.3:hlh-8p::nls::mCherry::lacZ]+ myo-2 ::mCherry]IV; egl-15 ( syb6504 )X *	Lo Lab
TWL34	*jjIs3900 [srsIm15.129pJKL1066.3:hlh-8p::nls::mCherry::lacZ]+ myo-2 ::mCherry]IV; egl-15 ( syb6343 )X *	Lo Lab
TWL35	*jjIs3900 [srsIm15.129pJKL1066.3:hlh-8p::nls::mCherry::lacZ]+ myo-2 ::mCherry]IV; egl-15 ( syb6294 )X *	Lo Lab
TWL36	*jjIs3900 [srsIm15.129pJKL1066.3:hlh-8p::nls::mCherry::lacZ]+ myo-2 ::mCherry]IV; egl-15 ( syb6346 )X *	Lo Lab
TWL37	*jjIs3900 [srsIm15.129pJKL1066.3:hlh-8p::nls::mCherry::lacZ]+ myo-2 ::mCherry]IV; egl-15 ( syb6335 )X *	Lo Lab
TWL38	*jjIs3900 [srsIm15.129pJKL1066.3:hlh-8p::nls::mCherry::lacZ]+ myo-2 ::mCherry]IV; egl-15 ( syb6438 )X *	Lo Lab
TWL39	*jjIs3900 [srsIm15.129pJKL1066.3:hlh-8p::nls::mCherry::lacZ]+ myo-2 ::mCherry]IV; egl-15 ( syb6431 )X *	Lo Lab
TWL40	*jjIs3900 [srsIm15.129pJKL1066.3:hlh-8p::nls::mCherry::lacZ]+ myo-2 ::mCherry]IV; egl-15 ( syb6350 )X *	Lo Lab
TWL41	*jjIs3900 [srsIm15.129pJKL1066.3:hlh-8p::nls::mCherry::lacZ]+ myo-2 ::mCherry]IV; egl-15 ( syb6624 )X *	Lo Lab

## References

[R1] Goodman SJ, Branda CS, Robinson MK, Burdine RD, Stern MJ. 2003. Alternative splicing affecting a novel domain in the C. elegans EGL-15 FGF receptor confers functional specificity. Development 130(16): 3757-66. 10.1242/dev.00604 12835392

[R2] Itoh N, Ornitz DM (2010). Fibroblast growth factors: from molecular evolution to roles in development, metabolism and disease.. J Biochem.

[R3] Lo TW, Branda CS, Huang P, Sasson IE, Goodman SJ, Stern MJ. 2008. Different isoforms of the C. elegans FGF receptor are required for attraction and repulsion of the migrating sex myoblasts. Dev Biol 318(2): 268-75. 10.1016/j.ydbio.2008.03.026 PMC2516447 18455716

[R4] Ornitz DM, Itoh N (2015). The Fibroblast Growth Factor signaling pathway.. Wiley Interdiscip Rev Dev Biol.

[R5] Sherwood DR, Plastino J (2018). Invading, Leading and Navigating Cells in Caenorhabditis elegans: Insights into Cell Movement in Vivo.. Genetics.

[R6] Stiernagle T (2006). Maintenance of C. elegans.. WormBook.

[R7] Sulston JE, Horvitz HR (1977). Post-embryonic cell lineages of the nematode, Caenorhabditis elegans.. Dev Biol.

[R8] Thomas JH, Stern MJ, Horvitz HR (1990). Cell interactions coordinate the development of the C. elegans egg-laying system.. Cell.

[R9] Trent C, Tsuing N, Horvitz HR (1983). Egg-laying defective mutants of the nematode Caenorhabditis elegans.. Genetics.

[R10] Shen Q, Shi H, Tian C, Ghai V, Liu J (2017). The C. elegans Spalt-like protein SEM-4 functions through the SoxC transcription factor SEM-2 to promote a proliferative blast cell fate in the postembryonic mesoderm.. Dev Biol.

